# Contributions of declining mortality, overall and from HIV, TB and malaria, to reduced health inequality and inequity across countries

**DOI:** 10.1093/heapol/czad046

**Published:** 2023-07-06

**Authors:** Markus Haacker

**Affiliations:** Heidelberg Institute of Global Health, University of Heidelberg, Heidelberg 69120, Germany; Center for Global Development, Washington, DC 20036, United States; Institute for Global Health, University College London, London WC1N 1EH, United Kingdom

**Keywords:** Inequality, HIV, tuberculosis, malaria, life expectancy

## Abstract

The objective to reduce global health inequalities and inequities is integral to the global development agenda, from the Universal Declaration of Human Rights to the sustainable development goals and the ongoing response to coronavirus disease. Yet, summary measures of global health gains or of the cost-effectiveness of global health programmes barely capture how well they improve the lives of the most disadvantaged populations. This paper instead explores the distribution of global health gains across countries and the implications for health inequality and inequity (here referring to health disadvantages that reinforce economic disadvantage, and vice versa) across countries. Specifically, it studies the distribution of gains in life expectancy across countries (overall and owing to reduced mortality from HIV, TB and malaria), using the Gini index and a concentration index ranking countries by gross domestic product (GDP) per capita as indicators of health inequality and inequity. By these counts, global inequality in life expectancy across countries declined by one-third between 2002 and 2019. Reduced mortality from HIV, TB and malaria accounted for one‐half of this decline. Fifteen countries in sub‐Saharan Africa, containing 5% of the global population, accounted for 40% of the global decline in inequality, with nearly six‐tenth of this contribution coming from HIV, TB and malaria. Inequity in life expectancy across countries declined by nearly 37%, with a contribution from HIV, TB and malaria of 39% of this gain. Our findings show how simple indicators on the distribution of health gains across countries usefully complement aggregate measures of global health gains and underscore their positive contribution to the global development agenda.

Key messages

- Global inequality in life expectancy across countries declined by one-third between 2002 and 2019. Reduced mortality from HIV, TB and malaria accounted for one‐half of this decline.
- Fifteeen countries in sub‐Saharan Africa, containing 5% of the global population, accounted for 40% of the global decline in inequality, with nearly six‐tenth of this contribution coming from HIV, TB and malaria.
- Simple indicators on the distribution of health gains across countries usefully complement aggregate measures of global health gains and underscore their positive contribution to the global development agenda.

## Introduction and purpose

The objective of reducing global health inequality and inequity is at the heart of and intertwined with the global development agenda. The ‘right to a standard of living adequate for the health and well-being of himself and of his family’ is enshrined in the Universal Declaration of Human Rights (United Nations General Assembly, 1948), while the Constitution of the World Health Organization declares that that ‘the enjoyment of the highest attainable standard of health is one of the fundamental rights of every human being without distinction of race, religion, political belief, economic or social condition’ (International Health Conference, 1946). The sustainable development goals (SDGs) embed health objectives under the ‘greatest global challenge’ of ‘eradicating poverty in all its forms and dimensions’ (United Nations General Assembly, 2015). Specific health goals are accordingly organized under the objectives of achieving ‘universal health coverage and access to quality health care’, as ‘no one must be left behind’. The Commission on Investing in Health (Jamison *et al.*, 2013) spelled out a pathway to achieving a ‘grand convergence’ in health and highlighted the economic returns of such health investments. Global health programmes are also embedded in the broader development context as funding almost always comes from government budgets for official development assistance. These programmes are thus accountable to funders on how well they advance the global development agenda and the objectives of funders’ development policies, which are often geared at countries and populations most behind in terms of health attainments or economic capacities.

Most recently, the experience of coronavirus disease has underscored the relevance of inequalities in health and access to health services both across and within countries and the interdependence of health and economic inequalities (Horton (2021), Gopalakrishnan et al. (2021)). Relatedly, the World Health Organization (WHO) (2021) has issued a comprehensive stocktaking of the evidence on socio-economic inequalities within countries with regard to HIV, TB and malaria, while the Joint United Nations Programme on HIV/AIDS (UNAIDS) has headlined inequality in the 2021 Global AIDS Update (UNAIDS, 2021) and its Global AIDS Strategy 2021–26 (UNAIDS, 2021b).

In contrast to this concern about inequality and its ethical underpinnings, common measures of effectiveness and cost-effectiveness applied in global health barely account for this development context and distributional concerns. Measures like life years gained, deaths averted or infections averted are neutral with regard to where the gains arise and how well the gains contribute to a convergence in health outcomes across countries. Disability-adjusted life years (DALYs), however, implicitly prioritize health gains in countries further behind because they adopt a benchmark of standardized life expectancy (derived using the globally lowest country-level overall mortality for each age group; see Roth and others, 2018). The lower actual life expectancy is against this benchmark, the higher is the gain in DALYs compared to the gain in life years. In contrast, estimates of ‘economic returns’ from investments in global health based on the value of statistical life or output gains, which result from longer survival, are dominated by gains in relatively wealthy countries as such valuations increase with GDP per capita. From this perspective, the more the health gains are concentrated in middle- rather than low-income countries, the higher is the ‘economic return’ from investing in global health (Lamontagne et al. (2019); Silva et al. (2021). If the value of the health gains is specified relative to GDP per capita, e.g. as contributions (alongside economic growth) to the growth of full-income (Crafts, 1997; Becker *et al.*, 2005; Jamison *et al.*, 2013), estimates of the contribution of health gains are broadly neutral with regard to the level of economic development.

The purpose of this paper is to explore how to complement these measures of the magnitude of health gains with indicators that provide insights into the effectiveness of observed health gains in improving outcomes in countries, which—in terms of life expectancy or GDP per capita—are furthest behind, and in reducing health inequality and inequity across countries.

To meet these objectives, we apply established summary indicators of the distributions of health outcome to gains in life expectancy across countries. Specifically, we use the Gini index [Atkinson (2013), Wagstaff et al. (1991)] to measure health inequality and a concentration index applying a ranking by GDP per capita [van Doorslaer and van Ourti (2011) or Wagstaff et al. (1991)] to measure health inequity (we use this term to refer to health disadvantages reinforcing economic disadvantage and vice versa). In addition, we look at the distribution of health gains across country income groupings (World Bank, 2021). While the cross-country perspective on global health inequality and inequity is partial (it does not capture within-country inequalities and systematic differences between population subgroups), it is attractive for our purposes because it gels with the country-level perspective of much of the programmatic statements on global health quoted earlier and their monitoring frameworks and with estimates on returns to investment in global health.

We apply these measures to assess gains in reducing global health inequality and inequity between 2002 and 2019, overall and with regard to the effects of reduced mortality from HIV, TB and malaria. The analysis covers a period characterized by a strong activist global health agenda (overall and for these three diseases), setting out from the United Nation’s ‘Millennium Declaration and Declaration of Commitment on HIV/AIDS’ (United Nations General Assembly, 2000; 2001), and the establishment of the Global Fund to Fight HIV, Tuberculosis, and Malaria (operating from 2002) and of the US President’s Emergency Plan for AIDS Relief (from 2003), which have become major players in global health financing.

Our study stands beside approaches like ‘extended cost-effectiveness analysis’, which includes the distribution of costs and benefits across socio-economic groups in the analysis (Verguet *et al.*, 2016), but—for better or worse—our approach differs from theirs as it uses a summary metre on the distribution of benefits and as we adopt a cross-country perspective. While inequalities in the burden of various diseases across countries have been observed before (e.g. Steinbeis *et al.*, 2019), we interpret trends in mortality changes (overall or from selected diseases) through the lens of an overall health outcome and thus apply an integrated perspective across diseases.

## Methods

We obtained overall and disease-specific mortality data from the Global Burden of Disease 2019 database (Institute of Health Metrics and Evaluation (IHME) (2020), and described by Vos and others (2020)). Specifically, we use country-level annual estimates of mortality by age group (<1 year, ages 1–4 years, ages 5–9 years and so on in 5-year intervals through ages 90–94 years and ages 95+ years), for all causes (Global Burden of Disease cause ID 169), owing to HIV (cause ID 298), tuberculosis (cause ID 297) or malaria (cause ID 345), for 2002 and 2019.

We calculate life expectancy from age-specific mortality rates using standard demographic methods (Preston *et al.*, 2001). While Institute of Health Metrics and Evaluation (IHME) (2020) also publish estimates of life expectancy, we do not use these Institute of Health Metrics and Evaluation (IHME) estimates as we will also calculate life expectancies for modified age profiles of mortality and require internal consistency between our estimates. Our calculated life expectancies, however, are very close to the IHME estimates (differing <0.2 years for almost all countries). These minor discrepancies likely reflect that IHME applies finer categories for infant mortality (breaking the 0–1 age range into three intervals) and that we might apply different assumptions on the distribution of mortality within age brackets, which is not fully documented by IHME.

To calculate the contributions of declining disease-specific mortality to changes in life expectancy over some period, we construct counterfactual age-specific mortality profiles applying the change in disease-specific mortality between 2002 and 2019 to overall mortality in 2002, obtaining a counterfactual overall mortality profile for 2019 as if only the disease-specific mortality had changed since 2002.

For example, all-cause mortality (*m*_all_) for age *a* in 2019, if only AIDS-related mortality (*m*_HIV_) had changed since 2002, would be equal to


(1) $$\begin{aligned}{m_{{\mathrm{all}},a,2002|{\mathrm{HI}}{{\mathrm{V}}_{2019}}}} & = {m_{{\mathrm{all}},a,2002}} + \left( {{m_{{\mathrm{HIV}},a,2019}} - {m_{{\mathrm{HIV}},a,2002}}} \right) \nonumber\\
&\quad\times \left[ {1 - \frac{{\left( {{m_{{\mathrm{all}},a,2002}} - {m_{{\mathrm{HIV}},a,2002}}} \right)}}{2}} \right].\end{aligned}$$

In this equation, the first term in brackets alone overwrites the disease- (here HIV-) specific mortality component in 2002 with the corresponding value for 2019. The second term makes an adjustment for non-HIV mortality as some people who die because of AIDS would otherwise die for different reasons. The alternate profile of mortality by age is then used to calculate the corresponding level of life expectancy. The combined contribution of HIV, TB and malaria to mortality is calculated similarly, adding up the changes in disease-specific mortality from the three diseases and otherwise applying the same method as described in Equation (1) for one disease. The estimates on life expectancy in 2002 and 2019, and the contributions of the three diseases, are documented in Annex Table 1 in the Supplementary Material for all countries.

While it is straightforward to add up changes in mortality from different sources, this does not extend to the resulting changes in life expectancy, because life expectancy is a non-linear function of mortality and changes in mortality from different sources interact with regard to the impacts on life expectancy. If mortality from one cause declines, the impact of a decline in mortality from another cause on life expectancy increases.

For this reason, the effect on life expectancy of combined changes in mortality from HIV, TB and malaria tends to be larger than the sum of the individual effects. These interaction effects are typically small (amounting to 0.7% of the combined effect on life expectancy on average) but can be higher (up to 7% of the total effect) in some countries with significant contributions from more than one of the three diseases (Annex Table 1 in the Supplementary Material). To address this inconsistency when decomposing total effects, we attribute the discrepancy arising from the interaction effect to each of the three diseases in proportion to their individual effects.

It is worth noting that for related reasons, our estimates of the impact of HIV, TB and malaria are conservative, as we use mortality profiles in 2002 as a benchmark for estimating the contributions of the three diseases to increases in life expectancy between 2002 and 2019. Because mortality from other causes has declined between 2002 and 2019, a similar calculation of the impacts of mortality from HIV, TB and malaria on life expectancy using 2019 data as a benchmark would yield higher estimates.

For aggregation and analysing the global distribution of health outcomes, we weigh country observations by population size, derived from Institute of Health Metrics and Evaluation (IHME) (2020). GDP per capita, used for calculating the concentration index, was obtained from International Monetary Fund (IMF) (2021). Estimates of GDP per capita were not available for various countries (Iraq, Nauru, San Marino, Somalia and South Sudan). These countries are excluded from estimates using GDP per capita, involving a small loss in population coverage (0.8% of the global population).

The Gini index of inequality is a tool that has commonly been used to analyse changes in inequality in incomes (Milanović, 2016). In this case, individuals or groups (here country populations) are first ordered by their level of income. The distribution on income can be represented by a Lorenz curve, which shows accumulated income in this ordering. The slope of the Lorenz curve is the income or the respective individual or group, and it is flat in the beginning (with low incomes) and successively becomes steeper for higher incomes. This is compared to a diagonal curve corresponding to perfect equality, the slope of which is average income across the population covered, which intersects with the Lorenz curve at the lowest and highest incomes. The more unequal the income distribution is, the further away is the Lorenz curve from the diagonal. The Gini index captures this distance, comparing the area between the Lorenz curve and the diagonal with the total area under the diagonal, and can take values between 0 (complete equality, Lorenz curve coincides with diagonal) and approaching 1 for extreme inequality.

The use of the Gini index in health economics is less common but is firmly established in the literature on health inequalities (Wagstaff et al. (1991), van Doorslaer and van Ourti (2011), Atkinson 2013)), as a comprehensive measure of inequality across the distribution of health outcomes. It is calculated in our study as described earlier, but measuring the distribution life expectancy instead of income and with populations ordered according to their level of life expectancy.

When interpreting health inequalities, it is important to keep in mind that these inequalities, as measured by the Gini index, do not compare well with measured income inequalities (Atkinson, 2013). This is because incomes between countries differ by a factor of ∼100 between some high-income countries and the least developed countries, whereas life expectancies differ by a factor of <2 (ranging from 52 to 84 years as of 2019). A difference in life expectancies between 52 and 84 years, however, is arguably more significant than a difference in incomes between, say, US$5200 and US$8400.

There are approaches to comparing inequalities in health and income or developing an integrated perspective on inequality (Becker *et al.*, 2005). As we focus on inequalities in health, such integrated approaches that also capture the evolving inequalities in income are out of bounds for this paper. We do, however, measure health inequity, in the sense of the extent to which low health attainments reinforce economic disadvantage, and vice versa, using the concentration index. This index has also been used in the analysis of health inequalities (van Doorslaer and van Ourti, 2011). It is calculated just like the Gini index, with the difference that populations are not ordered by the health outcome of interest but by an economic indicator, typically some measure of income or economic capacities. Specifically, we use GDP per capita in 2002, at the beginning of our study period, to order the country populations. If the orderings by life expectancy and GDP per capita coincide, the concentration index is equal to the Gini index. But the less the orderings align, the lower the concentration index is, and theoretically—if poorer countries tended to have higher live expectancy—it could even take negative values.

## Results

All estimates of country-level gains in life expectancy (overall, for each of the three diseases and their combined effect), as well as the data on GDP per capita and population size used in the calculations, are documented in the Annex (Supplementary Material). We present these country-level results in three steps. We first summarize the gains in life expectancy by country groups. We then discuss the distribution of life expectancy and of gains in life expectancy informally, drawing on some visual representations. Finally, we present findings on changes in health inequality and inequity across countries, using the Gini index and concentration index.

### Distribution of gains by country groupings

Table 1 describes gains in life expectancy by country groupings, applying the World Bank’s income classification for 2002 (World Bank, 2021), the beginning of our period of interest. For each group, gains in life expectancy are calculated as population-weighted average across its members. Because of their large individual weight, we report results for India (which was classified as a low-income country in 2002) and China (lower-middle-income country) separately and exclude these two countries from the group aggregates. We use beginning-of-period classifications as the classification at the end of the period is a result of economic developments within the period, which, in turn, could influence the health indicators we focus on—thus avoiding a form of selection bias. We are similarly interested in how well gains have benefitted countries where life expectancy has been low. To this end, we divide countries into countries with low, lower-middle, upper-middle, and high life expectancy, again separating out India (otherwise ‘low’) and China (otherwise ‘lower-middle’). Cut-offs between groups by life expectancy (at 67.5, 74 and 77 years) were chosen so that the groups are approximately of same size as the income groups.

**Table 1.** Gains in life expectancy, 2002–19, across country groups

	Gain in life expectancy (years)						
	Total	HIV, malaria and TB	HIV	Malaria	TB	HIV, malaria and TB (share of total gain, %)	Population (share, %)
	Countries grouped by income (2002 World Bank classification)						
Low-income countries	7.66	2.56	1.40	0.50	0.66	33.4	22.9
India	6.96	0.93	0.22	0.12	0.59	13.4	17.1
Lower-middle-income countries	4.56	0.44	0.28	0.01	0.15	9.6	18.3
China	5.59	0.10	−0.02	0.00	0.12	1.8	21.1
Upper-middle-income countries	2.26	0.15	0.10	0.00	0.05	6.9	5.3
High-income countries	2.61	0.05	0.04	0.00	0.02	2.1	15.4
Global	5.48	0.86	0.42	0.14	0.31	15.8	100.0
Share of gains accruing in low-income countries and India (percent)							
Low-income countries (excl. India)	32.0	67.9	76.9	84.0	48.8		
Low-income countries (incl. India)	53.8	86.4	86.0	99.1	81.4		
	Countries grouped by 2002 life expectancy						
Low life expectancy	8.79	2.85	1.70	0.53	0.62	32.4	21.6
India	6.96	0.93	0.22	0.12	0.59	13.4	17.1
Lower-middle life expectancy	3.51	0.29	0.05	0.01	0.23	8.2	19.4
China	5.59	0.10	−0.02	0.00	0.12	1.8	21.1
Upper-middle life expectancy	2.40	0.08	0.02	0.00	0.06	3.4	5.3
High life expectancy	2.60	0.06	0.04	0.00	0.02	2.1	15.6
Global	5.48	0.86	0.42	0.14	0.31	15.8	100.0
Share of gains accruing in countries with low life expectancy in 2002 and India (percent)							
Low life expectancy	34.6	71.2	87.9	83.6	43.3		
Low life expectancy and India	56.4	89.7	97.0	98.7	75.9		

Gains in life expectancy owing to reduced mortality from HIV, tuberculosis and malaria contributed about one-sixth (0.9 years out of 5.5 years) to global gains between 2002 and 2019 and one-third (2.6 years out of 7.6 years) to gains across low-income countries (excluding India). Low-income countries (including India) accounted for five-sixth of the global gains in life expectancy due to reduced mortality from HIV, TB and malaria, and gains from reduced mortality from HIV, TB and malaria have been 9.5 times stronger (relative to the population size) in low-income countries than elsewhere. While HIV makes the largest contribution overall of the three diseases, gains in reduced mortality from malaria have been most strongly concentrated in less economically developed countries where 99% of the gains occurred.

The picture is similar when countries are grouped by the initial level in life expectancy. Life expectancy has increased by 8.9 years across countries where it had been lowest in 2002, and gains in mortality from HIV, TB and malaria accounted for about one-third (2.9 years) of this gain. Overall, countries with low life expectancy initially contributed nine-tenth of the gains in life expectancy through reduced mortality from HIV, TB and malaria. Compared to the grouping by income, much of the difference in the role of HIV, TB and malaria comes from HIV, reflecting the situation in some middle-income countries with very high HIV prevalence and where life expectancy was depressed by over 10 or even up to 20 years as of 2002 and where the scaling-up of treatment has gone a long way in reversing this loss since then.

### Visual representations of distribution of health gains across countries

**Figure 1.**
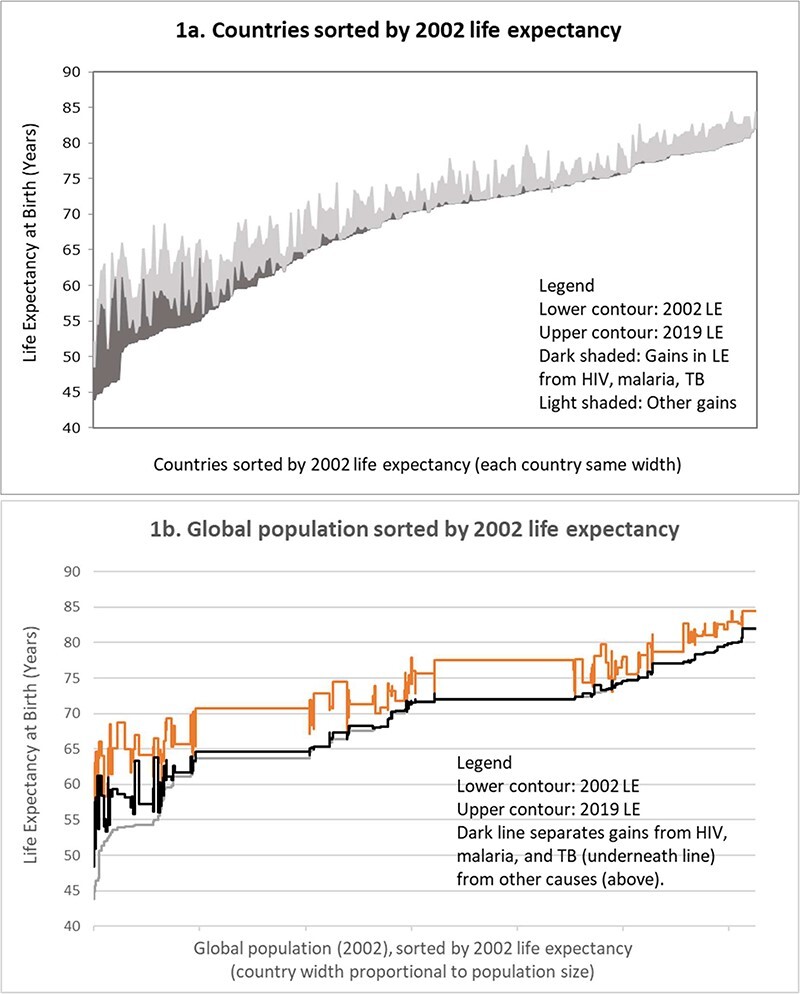
Gains in life expectancy, 2002–19, by 2002 life expectancy

**Figure 2.**
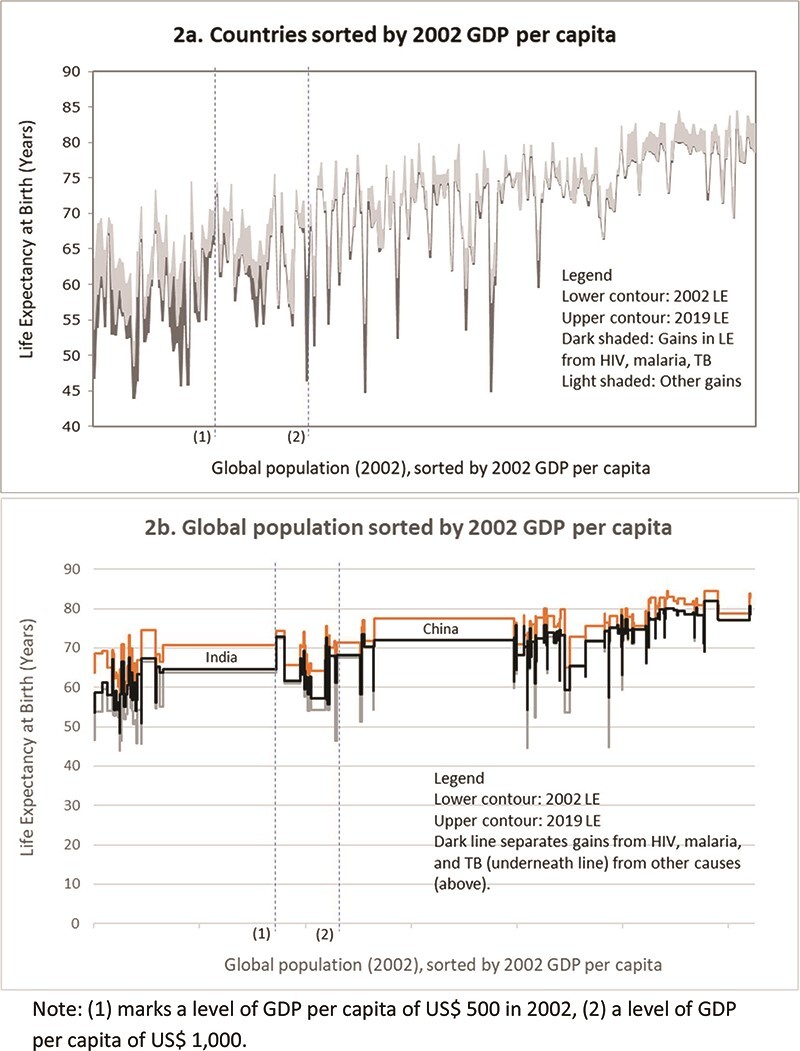
Gains in life expectancy, 2002–19, by 2002 GDP per capita

Note: (1) marks a level of GDP per capita of US$500 in 2002 and (2) a level of GDP per capita of US$1000.

Figures 1 and 2 provide a more complete picture of the gains in life expectancy across countries, illustrating gains since 2002, with countries and populations ordered by 2002 life expectancy (Figure 1) or 2002 GDP per capita (Figure 2). Gains attributed to reduced mortality from HIV, TB and malaria played a dominant role in a group of eight countries where life expectancy was lowest initially, at below 50 years in 2002 (Figure 1a), which, however, accounted for <1% of the global population (Figure 1b). In these countries, life expectancy increased from an average of 46 to 62 years. While the low level of life expectancy in 2002 underscores the devastating impacts of HIV, the gains since 2002 are dominated by the effects of the scaling-up of treatment for people living with HIV, which accounted for a gain in life expectancy of 10 years.

Outside this group of countries where initial life expectancy was lowest, gains on HIV, TB and malaria played a large role in countries where life expectancy was between 50 and 55 years in 2002 (23 countries, 9% of global population). In these countries, life expectancy increased by 11 years (from an average of 54 to 65 years). Reduced mortality from the three diseases played a crucial role here as well, accounting for nearly 5 years of gains (HIV 3 years, TB 1 year and malaria 0.8 years). HIV, TB and malaria still played an important role in countries starting out with a life expectancy between 55 and 60 years in 2002 (1.8 years out of total gains of 7.8 years). Beyond these countries, the contributions of reduced mortality from HIV, TB and malaria were much smaller (on average, 0.3 years out of total gains in life expectancy of 4.8 years), but there are some notable exceptions [Cambodia (2.5 years out of total gain of 8.4 years), India (0.9 years out of 7.0 years), Bangladesh (0.9 years out of 8.1 years), and Thailand (1.1 years out of 5.2 years)].

The impact of reduced mortality from HIV, TB and malaria on inequity across countries is less obvious visually (Figure 2). The gains are less concentrated on the left (among the countries where GDP per capita has been lowest), and there are significant contributions to life expectancy in a few countries with relatively high GDP per capita. But the underlying distribution of life expectancy is also less unequal than in Figure 1 (by construction as countries/populations are ordered by a variable other than life expectancy in Figure 2). The picture across countries, though, is consistent with the estimates by income group presented earlier. For example, contributions from HIV, TB and malaria (3.5 years out of a total of 9.7 years) were highest for countries that had a level of GDP per capita of up to US$500 in 2002 (excluding India; Figure 2b), which still made a very important contribution across countries with GDP per capita between US$500 and US$1000 in 2002 (1.6 years out of a total of 6.0 years), but were overall small in countries that had higher GDP per capita (0.3 years out of 4.3 years).

### Reduced health inequality and inequity

Summary indices like the Gini index or the concentration index across countries are attractive because they draw on the entire distribution of life expectancy across countries and are therefore not dependent on arbitrary groupings. However, unlike the estimates by country grouping, they do not come in natural units (here life expectancy) so are unsuitable to assess the absolute differences in the contribution to gains in life expectancy across countries.

Health inequality in life expectancy across countries, as measured by the Gini index has declined by one-third between 2002 and 2019 (Table 2), from 0.060 to 0.040. Reduced mortality from HIV, TB and malaria made a large contribution, accounting for one-half of the overall decline in health inequality. Reduced AIDS-related mortality accounted for more than one-half of the contribution from the three diseases and 27.5% of the total decline in inequality in life expectancy across countries. Health inequity in life expectancy across countries, as measured by the concentration index (ranking countries by GDP per capita), has declined similarly by 37.1% between 2002 and 2019. Declining AIDS-related mortality contributed 38.8% of this decline in health inequity, and HIV in turn about one-half of the contribution from the three diseases.

**Table 2.** Contributions of mortality gains linked to HIV, TB and malaria to declining inequality and inequity in life expectancy across countries

	Inequality			
Gini index in 2002	0.060		Absolute change	−0.019
Gini index in 2019	0.040		Relative change (%)	−32.6
Contributions from HIV, malaria and TB				
	All three	HIV	TB	Malaria
All countries				
Contribution to change	−0.009	−0.005	−0.002	−0.002
Percent of total gain	48.6	27.5	12.4	9.0
			**Inequity**	
Concentration index in 2002	0.050		Absolute change	−0.018
Concentration index in 2019	0.031		Relative change (%)	−37.1
Contributions from HIV, malaria and TB				
	All three	HIV	TB	Malaria
All countries				
Contribution to change	−0.007	−0.004	−0.002	−0.001
Percent of total gain	38.8	19.2	12.5	7.4

Note: Because the Gini index and the concentration index are non-linear functions, the results on HIV, TB and malaria do not precisely add up to the combined effect.

These summary indices provide information on how effective declines in mortality from HIV, TB and malaria have been overall in improving health outcomes in countries, which were behind in terms of life expectancy or in countries where economic disadvantage (in the form of low GDP per capita) was compounded by low life expectancy. The approach, however, also yields insights into the roles of individual countries.

The largest contribution to reduced global inequality in life expectancy was from India (17% of the global population, life expectancy rising from 63.7 years in 2002 to 70.7 years in 2019), which alone contributed 38% to the global decline in health inequality, with a relatively small contribution (one-sixth) from HIV, TB and malaria (Annex Table 3 in the Supplementary Material). (In contrast, the gains in China—starting at a much higher level of life expectancy—contributed a small increase to inequality in life expectancy across countries.) The most effective contributions (relative to country size) came from a group of countries, all in sub-Saharan Africa, which started out with life expectancy below or just above 50 years, have experienced large gains in life expectancy, where gains in life expectancy were dominated by HIV, TB and malaria (Annex Table 4 in the Supplementary Material). The strongest contributor from this perspective was Malawi, where the life expectancy increased from 46.3 years in 2002 to 64.6 years in 2019. While Malawi accounts for only 0.2% of the global population, it contributed 2.3% to the decline in health inequality across countries. Fifteen countries in sub-Saharan Africa (Annex Table in the Supplementary Material), with 5% of the global population and life expectancy on average increasing from 52.3 years to 65.7 years, accounted for 40% of the global decline in inequality. Reduced mortality from HIV, TB and malaria accounted for nearly six-tenth of the contribution from these 15 countries (and thus 24% of the global decline in inequality).

As it was for inequality, the largest contribution to reduced health inequity again was from India, which alone accounted for 62% of the global decline in inequity (but little of this came from the three diseases). From the perspective of health equity, the relatively large health gain was reinforced here by a low starting point for GDP per capita. The strongest contribution, relative to country size, came from Burundi, where GDP per capita was extremely low (US$116 in 2002), which contributed 1.5% of the global decline in health inequity with a population size corresponding to 0.1% of the global population. Fifteen countries accounting for 5.2% of the global population accounted for 52% of the global decline in health inequity across countries. Declining mortality from HIV, TB and malaria in these 15 countries alone accounted for a decline in health inequity across countries of 23%, equivalent to nearly one-half (45%) of the contribution from these countries.

Only about one-half of countries (8 out of 15) contributing most strongly to declining health inequality across countries also appear among the countries making the strongest contributions to declining health inequity across countries. HIV, TB and malaria play a large role across the groupings in Table 3 on aggregate, accounting for about one-half or more of the gains in life expectancy. The strongest contributors to health inequity (but not health inequality), though, differ from the other countries listed in Table 3 in two regards. It also includes countries from outside sub-Saharan Africa, and it includes several countries (Guinea-Bissau, Laos, Liberia, Myanmar and Niger) where HIV, TB and malaria accounted for only between one-eighth and one-quarter of the gain in life expectancy.

**Table 3.** Countries providing strongest contributions to declining health inequality and inequity across countries, 2002–19

Both health inequality and inequity	Health inequality only	Health inequity only
Burundi, Democratic Republic of Congo, Ethiopia, Malawi, Rwanda, Tanzania, Uganda, Zambia	Botswana, Central African Republic, Côte d’Ivoire, Eswatini, Namibia, South Africa, Zimbabwe	Guinea-Bissau, Kenya, Laos, Liberia, Myanmar, Niger, Sierra Leone

‘Strongest’ contribution = largest contribution relative to population size.

## Discussion

This paper set out to explore the use of summary indicators of health inequality and inequity to assess gains in global health outcomes, overall and with a focus on HIV, TB and malaria, three diseases that played a large role in the global health agenda over the last two decades. The analysis serves two objectives, one methodological and one practical: (1) to provide a contribution on complementing the most common measures used in economic evaluations of global health programmes, which either are neutral with regard to location of gains (e.g. deaths averted) or prioritize gains in countries with higher income (through assessed ‘productivity’ or ‘full-income’ gains), with an indicator that reflects how well gains benefit countries furthest behind (in terms of health attainments or economic capacities), and (2) to provide insights on how effective health gains achieved over the last two decades have been in improving living conditions in the most disadvantaged countries.

From the latter perspective, the period from 2002 to 2019 was characterized by strong gains in life expectancy in countries where this had been lowest or where low life expectancy coincided with a low level of economic development. For example, inequality in life expectancy across countries (measured by the Gini index) declined by one-third over this period, and reduced mortality from HIV, TB, and malaria accounted for one-half of this gain. We also show that gains in some of the countries furthest behind have contributed very effectively to improving the global picture on inequality and inequity (Annex Table 4 in the Supplementary Material).

While the Gini index and the concentration index are useful tools to summarize degrees of inequality and inequity across countries, it is important to note that they are ad hoc measures and not supported by an explicit value framework. However, the two measures give preference to health gains in countries further behind and are thus linked to the idea of a human ‘right to a standard of living adequate for the health and well-being’ (United Nations General Assembly, 1948), the goal of the SDGs of ‘eradicating poverty in all its forms and dimensions’ (United Nations General Assembly, 2015), or to conceptions of social justice focusing on the least well-off (Rawls, 1971) and of health inequity and fairness (Norheim and Asada, 2009; Ottersen and Norheim, 2014). In this sense, the focus on the countries furthest behind implied by the indicators on health inequality and inequity we apply provides a valid addition and counterpoint to headcount indicators (deaths averted, lives saved, etc.), which value health gains equally irrespective of location and economic circumstances. Our indicators contrast with estimates of ‘economic gains’, which value health gains in line with GDP per capita or income. These economic valuations may have stronger microeconomic and empirical foundations, but their application to global health programmes is problematic as these ‘economic gains’ are driven by outcomes in the relatively wealthy countries and thus run against the focus on reducing poverty and improving outcomes for the least well-off.

As we are focusing on inequality and inequity across countries, we miss out on inequality and socio-economic disparities within countries. This focus can be considered legitimate from a pragmatic perspective, as we are adopting a global health perspective, and evaluations of global health programmes largely focus on national-level data. However, from human rights or poverty reduction perspectives, a focus on national-level data can be insufficient if it does not capture barriers in access to health services and disparities in health outcomes within countries. In this regard, the picture is ambiguous with regard to HIV, TB and malaria. World Health Organization (WHO) (2021) highlights persistent inequities in access to health services on HIV, TB and malaria. On the other hand, the large reductions in mortality from HIV, TB and mortality and resulting gains in life expectancy reflect steep increases in the coverage of health services, and it is plausible that these have contributed to reduced health inequity within countries. More generally, life expectancy can be considered an indicator of health inequity to the extent that it reflects the consequences of premature mortality or mortality from causes avoidable by those who can afford access to an adequate package of health services (Jamison, 2020).

## Conclusions

We propose that estimates of changes in health inequality and inequity across countries—measured by the Gini index or the concentration index, respectively—provide important insights into global health gains and usefully complement commonly used indicators like life years saved or economic returns. In contrast to these latter indicators, which are largely neutral to where gains occur or prioritize more advanced economies, health gains that contribute to reduced health inequality and inequity are disproportionately effective in improving health outcomes for populations further behind.

The usefulness of considering indicators of health inequality in global health is demonstrated with an analysis of health gains in 2002–19, overall and owing to reduced mortality from HIV, TB and malaria, which played a large role in the global health agenda over this period. While gains on HIV, TB and malaria contributed about one-sixth of global gains in life expectancy over this period, this gain accounted for one-half of the global decline in inequality in life expectancy across countries over this period. Moreover, our analysis documents the effectiveness in reducing global health inequality and inequity of improved health outcomes in some of the countries furthest behind—gains in 15 countries comprising 5% of the global population accounted for 40% of the global decline in inequality in life expectancy across countries.

## Supplementary Material

czad046_Supp

## Data Availability

The paper uses data which are in the public domain. This is documented in the text: “We obtained overall and disease-specific mortality data from the Global Burden of Disease 2019 database (Institute of Health Metrics and Evaluation (IHME) (2020)).” I understand that this statement is sufficient on data availability. The text provides further information on which data specifically has been obtained and how this data has been used.

## Reflexivity statement

The paper is a single-author piece that builds on publicly available global data. Statements on the situation in any particular country or region are confined to descriptions of the contributions to global health trends building on Global Burden of Disease 2019 estimates and do not include discussions on health systems challenges which would require more definite regional or country-level expertise.
