# Supplementary material for: Contributions of declining mortality, overall and from HIV, TB and malaria, to reduced health inequality and inequity across countries
Source: Health Policy Plan. 2023 Jul 6;38(8):939–48. doi: 10.1093/heapol/czad046 (PMC10506528; doi:10.1093/heapol/czad046)
Supplement: czad046_Supp [file czad046_supp.zip › suppl_data/global-health-inequality-hpp-r2-annex.docx]

# Data Annex

[To be presented online as supplementary material.]

Annex Table 1 contains the full data set used in the analysis on contributions of changes in life expectancy (overall and owing to declining mortality from HIV, TB, and malaria) to declining health inequality (in life expectancy) across countries:

- Population (millions, 2002), based on IHME (2020). This is used for weighing countries in the calculation of the Gini and concentration indices, and for identifying countries which make a large contribution to declining health inequality relative to their country size. We use 2002 population weights throughout to focus on the contribution of changes in mortality on health inequality across countries, excluding the (small) effects of changes in relative population sizes.
- GDP per capita (in US$, 2002), from IMF (2021). This is used for ordering countries in the calculation of the concentration index. We use 2002 GDP per capita throughout to focus on the contribution of changes in mortality on health inequality across countries, excluding the (small) effects of changes in the ranking of countries by GDP per capita between 2002 and 2019.
- Estimates on life expectancy in 2002 and 2019, and or the gain between 2002 and 2019. This has been calculated based on age-specific mortality estimates for 2002 and 2019, respectively (from IHME, 2020), using standard demographic methods (Preston, Heuveline, and Guillot, 2001), and validated against estimates of life expectancy obtained directly from IHME (2020). We calculate estimates of life expectancy rather than using the IMHE estimates directly to ensure consistency with our estimates of life expectancy based on variations in mortality profiles.
- Estimates of the impact of changes in mortality from HIV, TB, malaria and the three diseases combined (labelled HTM) on life expectancy between 2002 and 2019. This has been calculated using the 2002 mortality profiles by age as a starting point, and adjusting them by the change in mortality from the respective disease(s), as documented in Eq. 1 in the paper. In using and interpreting these data, it is important to take into account that the impacts of mortality declines from one disease and another disease interact with regard to impacts on life expectancy. Lower mortality from one cause means that the impact on life expectancy from changes in mortality from another cause increases. This is evident in Annex Table 1 because the impacts of declining mortality from the three diseases combined (e.g., 9.02 years in Tanzania) exceed the sum of the individual contributions from HIV, TB, and malaria (7.09 years, 1.06 years, and 0.50 years, summing up to 8.65 years) by an interaction effect amounting to 0.37 years. To obtain consistent estimates for HIV, TB, malaria, and the combined effect, we attribute the interaction effect for each country to the three diseases in proportion to their respective individual effects. E.g., for Tanzania, the effect of HIV, TB, and malaria, including this attribution, are 7.40 years, 1.10 years, and 0.52 years.
- The Gini indices on life expectancy across countries for 2002 and 2019 (0.060 and 0.040, respectively) were calculated based on the profile of life expectancy for each year, using 2002 population levels as weights. The contribution of individual countries was calculated by substituting the 2019 level of life expectancy for that country for the 2002 level, and re-calculating the Gini index with all other parameters unchanged. Because the changes in the Gini index attributable to increased life expectancy in individual countries are quite small in absolute terms, we show the contribution in percent of the total change in the Gini index between 2002 and 2019.

In addition to the summary table (Annex Table 1) presenting all data used in the analysis on changes in life expectancy across countries, we present 3 tables highlighting various aspects of the data:

- Annex Table 2 shows the data and results for the 15 countries which have experienced the largest overall increase in life expectancy between 2002 and 2019.
- Annex Table 3 shows the data and results for the 15 countries which made the largest contribution to reducing global inequality in life expectancy between 2002 and 2019.
- Annex Table 4 shows the data and results for the 15 countries which made the largest contribution to reducing global inequality in life expectancy between 2002 and 2019, relative to the population size of the respective country.

| **Annex Table1. Contribution of Reduced Mortality from HIV, TB, and Malaria to Increasing Life Expectancy, 2002-2019** | | | | | | | | | | |
| --- | --- | --- | --- | --- | --- | --- | --- | --- | --- | --- |
|  | Population (millions) | GDP per capita (US$) | Life expectancy (Years) | | | Contribution to Gain in Life Expectancy (Years) | | | | Contribution to Gini (%) |
| Country | 2002 | 2002 | 2002 | 2019 | Gain | HTM | HIV | TB | Malaria |  |
| Afghanistan | 21.5 | 233 | 55.5 | 63.1 | 7.6 | 0.55 | -0.01 | 0.24 | 0.31 | 1.6 |
| Albania | 3.1 | 1,425 | 75.7 | 78.2 | 2.6 | 0.01 | 0.00 | 0.00 | 0.01 | -0.1 |
| Algeria | 32.0 | 1,807 | 71.5 | 76.1 | 4.5 | 0.03 | 0.00 | 0.00 | 0.03 | -0.8 |
| Angola | 16.0 | 841 | 54.1 | 65.0 | 10.8 | 1.65 | -0.36 | 0.66 | 1.35 | 1.6 |
| Antigua and Barbuda | 0.1 | 10,482 | 75.2 | 76.2 | 0.9 | 0.20 | 0.19 | 0.00 | 0.01 | 0.0 |
| Argentina | 37.6 | 2,998 | 74.2 | 76.4 | 2.3 | 0.07 | 0.04 | 0.00 | 0.02 | -0.6 |
| Armenia | 3.3 | 779 | 72.5 | 75.6 | 3.1 | 0.05 | -0.01 | 0.00 | 0.06 | -0.1 |
| Australia | 19.3 | 21,648 | 80.1 | 82.7 | 2.6 | 0.01 | 0.01 | 0.00 | 0.00 | -0.5 |
| Austria | 8.1 | 26,508 | 78.9 | 82.0 | 3.1 | 0.01 | 0.01 | 0.00 | 0.00 | -0.3 |
| Azerbaijan | 8.4 | 761 | 66.7 | 70.8 | 4.1 | 0.25 | 0.01 | 0.00 | 0.24 | 0.1 |
| Bahamas, The | 0.3 | 28,477 | 71.8 | 73.2 | 1.4 | 1.03 | 1.02 | 0.00 | 0.02 | 0.0 |
| Bahrain | 0.7 | 13,502 | 72.6 | 76.9 | 4.3 | 0.05 | 0.02 | 0.00 | 0.03 | 0.0 |
| Bangladesh | 131.1 | 421 | 66.4 | 74.5 | 8.1 | 0.92 | 0.00 | 0.17 | 0.76 | -0.1 |
| Barbados | 0.3 | 11,443 | 75.0 | 76.1 | 1.1 | 0.29 | 0.29 | 0.00 | 0.00 | 0.0 |
| Belarus | 10.1 | 1,524 | 68.0 | 73.9 | 5.9 | 0.06 | -0.02 | 0.00 | 0.08 | 0.0 |
| Belgium | 10.3 | 25,044 | 78.3 | 81.2 | 2.9 | 0.02 | 0.01 | 0.00 | 0.01 | -0.3 |
| Belize | 0.3 | 3,518 | 71.3 | 74.3 | 3.0 | 0.44 | 0.35 | 0.00 | 0.08 | 0.0 |
| Benin | 7.2 | 575 | 57.7 | 64.3 | 6.6 | 1.90 | 1.12 | 0.38 | 0.37 | 0.4 |
| Bhutan | 0.7 | 813 | 67.3 | 72.9 | 5.7 | 0.97 | -0.02 | 0.48 | 0.51 | 0.0 |
| Bolivia | 8.7 | 915 | 67.3 | 71.9 | 4.5 | 0.54 | 0.04 | 0.00 | 0.49 | 0.1 |
| Bosnia and Herzegovina | 4.1 | 1,785 | 75.0 | 76.9 | 1.9 | 0.04 | 0.00 | 0.00 | 0.04 | -0.1 |
| Botswana | 1.7 | 3,018 | 44.9 | 62.2 | 17.3 | 12.52 | 11.73 | 0.00 | 0.44 | 0.3 |
| Brazil | 179.2 | 2,856 | 71.6 | 75.7 | 4.1 | 0.13 | 0.05 | 0.01 | 0.06 | -3.8 |
| Brunei Darussalam | 0.3 | 18,786 | 72.8 | 74.2 | 1.4 | 0.05 | -0.01 | 0.00 | 0.06 | 0.0 |
| Bulgaria | 7.8 | 2,091 | 70.6 | 73.3 | 2.7 | 0.02 | 0.00 | 0.00 | 0.02 | 0.0 |
| Burkina Faso | 13.1 | 294 | 53.3 | 61.5 | 8.2 | 5.04 | 2.36 | 2.40 | 0.08 | 1.1 |
| Burundi | 6.6 | 116 | 46.7 | 63.7 | 17.0 | 6.93 | 3.93 | 1.75 | 0.82 | 1.2 |
| Cabo Verde | 0.5 | 1,502 | 70.6 | 73.6 | 3.0 | 1.12 | 0.86 | -0.05 | 0.30 | 0.0 |
| Cambodia | 13.0 | 338 | 61.3 | 69.8 | 8.4 | 2.53 | 1.26 | 0.10 | 1.12 | 0.6 |
| Cameroon | 16.2 | 708 | 54.1 | 63.2 | 9.2 | 4.19 | 2.56 | 1.04 | 0.46 | 1.5 |
| Canada | 30.8 | 24,279 | 79.6 | 81.9 | 2.3 | 0.02 | 0.02 | 0.00 | 0.00 | -0.8 |
| Central African Republic | 3.8 | 261 | 43.9 | 52.1 | 8.3 | 4.59 | 3.38 | 0.57 | 0.49 | 0.4 |
| Chad | 8.9 | 250 | 52.4 | 60.2 | 7.8 | 1.86 | 0.93 | 0.29 | 0.61 | 0.7 |
| Chile | 15.5 | 4,444 | 77.2 | 80.0 | 2.8 | 0.07 | 0.02 | 0.00 | 0.05 | -0.4 |
| China | 1,316.4 | 1,141 | 71.9 | 77.5 | 5.6 | 0.10 | -0.02 | 0.00 | 0.12 | -30.3 |
| Colombia | 41.2 | 2,355 | 73.1 | 79.8 | 6.7 | 0.15 | 0.04 | 0.07 | 0.05 | -2.2 |
| Comoros | 0.6 | 757 | 62.1 | 68.5 | 6.4 | 1.03 | 0.00 | 0.33 | 0.70 | 0.0 |
| Congo, Dem. Rep. | 54.2 | 171 | 54.0 | 64.9 | 10.9 | 4.11 | 1.68 | 1.39 | 0.91 | 5.6 |
| Congo, Rep. | 3.4 | 1,109 | 54.4 | 65.3 | 11.0 | 5.05 | 3.49 | 0.68 | 0.71 | 0.3 |
| Costa Rica | 4.0 | 4,123 | 78.3 | 79.8 | 1.5 | 0.07 | 0.03 | 0.00 | 0.04 | -0.1 |
| Côte d'Ivoire | 18.1 | 1,010 | 51.3 | 64.0 | 12.7 | 7.02 | 5.17 | 1.11 | 0.44 | 2.4 |
| Croatia | 4.5 | 6,229 | 75.0 | 78.5 | 3.6 | 0.03 | 0.00 | 0.00 | 0.03 | -0.1 |
| Cyprus | 1.0 | 16,189 | 77.2 | 80.7 | 3.5 | 0.00 | 0.00 | 0.00 | 0.01 | 0.0 |
| Czech Republic | 10.2 | 8,056 | 75.6 | 79.3 | 3.7 | 0.00 | 0.00 | 0.00 | 0.01 | -0.3 |
| Denmark | 5.4 | 33,276 | 77.3 | 80.9 | 3.7 | 0.01 | 0.01 | 0.00 | 0.01 | -0.2 |
| Djibouti | 0.7 | 1,241 | 59.9 | 66.6 | 6.7 | 1.82 | 0.95 | -0.09 | 0.94 | 0.0 |
| Dominica | 0.1 | 4,680 | 72.2 | 72.2 | 0.0 | 0.15 | 0.13 | 0.00 | 0.03 | 0.0 |
| Dominican Republic | 8.9 | 3,158 | 73.6 | 73.0 | -0.6 | 0.96 | 0.92 | 0.00 | 0.04 | 0.0 |
| Ecuador | 12.8 | 2,180 | 74.0 | 76.3 | 2.3 | 0.22 | -0.06 | 0.10 | 0.19 | -0.2 |
| Egypt, Arab Rep. | 70.6 | 1,355 | 68.1 | 70.9 | 2.8 | 0.02 | 0.00 | 0.00 | 0.02 | 0.4 |
| El Salvador | 5.9 | 2,124 | 74.0 | 75.5 | 1.5 | 0.09 | 0.06 | 0.00 | 0.03 | -0.1 |
| Equatorial Guinea | 0.7 | 3,108 | 59.9 | 65.9 | 6.0 | 1.23 | -0.89 | 1.36 | 0.80 | 0.0 |
| Eritrea | 4.5 | 227 | 56.5 | 63.7 | 7.1 | 2.57 | 1.45 | 0.01 | 1.05 | 0.3 |
| Estonia | 1.4 | 5,362 | 71.3 | 77.9 | 6.6 | 0.03 | -0.06 | 0.00 | 0.09 | -0.1 |
| Eswatini | 1.0 | 1,474 | 44.8 | 58.1 | 13.3 | 9.70 | 8.87 | 0.02 | 0.55 | 0.2 |
| Ethiopia | 71.2 | 116 | 53.9 | 68.7 | 14.8 | 4.69 | 2.72 | 0.31 | 1.47 | 8.4 |
| Fiji | 0.8 | 2,468 | 66.5 | 68.3 | 1.8 | 0.05 | 0.01 | 0.00 | 0.04 | 0.0 |
| Finland | 5.2 | 27,008 | 78.4 | 81.7 | 3.2 | 0.02 | 0.00 | 0.00 | 0.01 | -0.2 |
| France | 60.7 | 25,137 | 79.4 | 82.6 | 3.3 | 0.06 | 0.04 | 0.00 | 0.02 | -2.1 |
| Gabon | 1.3 | 4,210 | 59.6 | 67.7 | 8.1 | 3.83 | 2.56 | 0.60 | 0.56 | 0.1 |
| Gambia, The | 1.4 | 632 | 61.2 | 66.6 | 5.4 | 2.36 | 0.27 | 1.82 | 0.24 | 0.1 |
| Georgia | 4.5 | 875 | 70.3 | 73.3 | 3.0 | 0.06 | -0.01 | 0.00 | 0.08 | 0.0 |
| Germany | 82.5 | 25,460 | 78.6 | 81.0 | 2.4 | 0.01 | 0.01 | 0.00 | 0.00 | -2.0 |
| Ghana | 20.2 | 714 | 59.4 | 66.2 | 6.8 | 3.22 | 1.51 | 1.14 | 0.49 | 1.1 |
| Greece | 11.2 | 14,066 | 78.8 | 80.8 | 2.0 | 0.00 | 0.00 | 0.00 | 0.00 | -0.2 |
| Grenada | 0.1 | 5,214 | 72.0 | 73.2 | 1.2 | 0.10 | 0.10 | 0.00 | 0.01 | 0.0 |
| Guatemala | 11.6 | 1,796 | 67.7 | 72.5 | 4.8 | 0.16 | 0.06 | 0.01 | 0.09 | 0.1 |
| Guinea | 8.3 | 445 | 54.7 | 61.1 | 6.4 | 1.47 | 0.32 | 0.84 | 0.30 | 0.5 |
| Guinea-Bissau | 1.3 | 375 | 51.7 | 60.8 | 9.1 | 2.32 | 0.87 | 0.79 | 0.61 | 0.1 |
| Guyana | 0.8 | 2,093 | 63.8 | 67.2 | 3.4 | 0.95 | 0.68 | 0.16 | 0.11 | 0.0 |
| Haiti | 8.6 | 698 | 57.9 | 63.6 | 5.7 | 3.02 | 2.74 | 0.12 | 0.14 | 0.5 |
| Honduras | 6.5 | 1,122 | 70.3 | 72.0 | 1.7 | 0.20 | 0.09 | 0.00 | 0.10 | 0.0 |
| Hungary | 10.2 | 6,645 | 72.5 | 76.5 | 3.9 | 0.03 | 0.02 | 0.00 | 0.01 | -0.3 |
| Iceland | 0.3 | 32,516 | 80.6 | 83.8 | 3.1 | 0.02 | 0.01 | 0.00 | 0.01 | 0.0 |
| India | 1,070.5 | 479 | 63.7 | 70.7 | 7.0 | 0.93 | 0.22 | 0.12 | 0.59 | 38.4 |
| Indonesia | 219.2 | 1,003 | 67.6 | 71.3 | 3.7 | 0.64 | -0.04 | 0.01 | 0.67 | 1.9 |
| Iran, Islamic Rep. | 69.2 | 1,948 | 72.3 | 77.7 | 5.3 | 0.00 | -0.02 | 0.00 | 0.02 | -2.5 |
| Iraq | 28.6 | n.a. | 69.2 | 73.1 | 3.9 | 0.12 | 0.00 | 0.00 | 0.12 | -0.1 |
| Ireland | 4.0 | 32,489 | 77.7 | 81.8 | 4.2 | 0.01 | 0.00 | 0.00 | 0.01 | -0.2 |
| Israel | 6.7 | 18,489 | 78.7 | 82.8 | 4.0 | 0.01 | 0.00 | 0.00 | 0.01 | -0.3 |
| Italy | 57.1 | 22,384 | 80.0 | 82.9 | 2.9 | 0.03 | 0.02 | 0.00 | 0.01 | -1.8 |
| Jamaica | 2.7 | 3,710 | 75.4 | 76.0 | 0.7 | 0.38 | 0.37 | 0.00 | 0.01 | 0.0 |
| Japan | 129.5 | 32,832 | 81.9 | 84.4 | 2.5 | 0.03 | 0.00 | 0.00 | 0.03 | -3.6 |
| Jordan | 4.9 | 1,869 | 73.2 | 78.1 | 4.9 | 0.02 | 0.00 | 0.00 | 0.02 | -0.2 |
| Kazakhstan | 15.0 | 1,657 | 64.6 | 71.9 | 7.2 | 0.48 | 0.00 | 0.00 | 0.47 | 0.3 |
| Kenya | 32.8 | 475 | 55.0 | 66.4 | 11.4 | 8.85 | 7.78 | 0.43 | 0.39 | 3.2 |
| Kiribati | 0.1 | 825 | 56.9 | 60.7 | 3.8 | 0.36 | 0.01 | 0.00 | 0.36 | 0.0 |
| Korea, Rep. | 47.2 | 13,160 | 77.2 | 82.7 | 5.5 | 0.13 | 0.00 | 0.00 | 0.13 | -2.6 |
| Kuwait | 2.0 | 15,759 | 78.3 | 81.2 | 2.9 | 0.05 | 0.00 | 0.00 | 0.05 | -0.1 |
| Kyrgyz Republic | 5.1 | 323 | 67.2 | 73.3 | 6.1 | 0.32 | 0.01 | 0.00 | 0.32 | 0.0 |
| Lao PDR | 5.6 | 336 | 59.5 | 68.8 | 9.3 | 1.15 | -0.06 | 0.07 | 1.14 | 0.3 |
| Latvia | 2.3 | 4,116 | 70.4 | 75.7 | 5.3 | 0.02 | -0.06 | 0.00 | 0.07 | -0.1 |
| Lebanon | 3.8 | 4,503 | 75.0 | 76.5 | 1.5 | 0.04 | 0.02 | 0.00 | 0.01 | 0.0 |
| Lesotho | 2.0 | 410 | 45.8 | 51.7 | 5.9 | 5.24 | 5.08 | 0.00 | 0.13 | 0.1 |
| Liberia | 3.0 | 336 | 54.8 | 65.9 | 11.1 | 2.84 | 0.86 | 1.57 | 0.35 | 0.3 |
| Libya | 5.3 | 3,834 | 74.9 | 75.8 | 0.9 | 0.01 | -0.01 | 0.00 | 0.02 | 0.0 |
| Lithuania | 3.4 | 4,141 | 71.9 | 76.1 | 4.2 | 0.08 | -0.01 | 0.00 | 0.09 | -0.1 |
| Luxembourg | 0.4 | 53,399 | 78.5 | 82.7 | 4.2 | 0.02 | 0.01 | 0.00 | 0.00 | 0.0 |
| Madagascar | 16.8 | 319 | 59.8 | 65.3 | 5.6 | 0.88 | -0.11 | 0.46 | 0.53 | 0.8 |
| Malawi | 11.6 | 273 | 46.4 | 64.6 | 18.3 | 11.82 | 9.75 | 0.87 | 0.58 | 2.3 |
| Malaysia | 24.9 | 4,442 | 73.4 | 74.9 | 1.5 | 0.22 | 0.09 | 0.01 | 0.12 | -0.2 |
| Maldives | 0.3 | 2,952 | 73.6 | 78.9 | 5.3 | 0.21 | 0.00 | 0.00 | 0.21 | 0.0 |
| Mali | 11.9 | 346 | 54.1 | 61.6 | 7.6 | 2.67 | 0.75 | 1.53 | 0.35 | 0.9 |
| Malta | 0.4 | 11,346 | 79.0 | 82.3 | 3.4 | 0.01 | 0.01 | 0.00 | 0.00 | 0.0 |
| Marshall Islands | 0.1 | 2,651 | 62.8 | 65.4 | 2.5 | 0.13 | -0.07 | 0.00 | 0.20 | 0.0 |
| Mauritania | 2.7 | 635 | 64.4 | 70.7 | 6.3 | -0.23 | 0.04 | -0.63 | 0.38 | 0.1 |
| Mauritius | 1.2 | 4,196 | 72.5 | 75.3 | 2.8 | -0.13 | -0.13 | 0.00 | 0.00 | 0.0 |
| Mexico | 103.9 | 7,607 | 74.6 | 75.6 | 1.0 | 0.10 | 0.04 | 0.00 | 0.05 | -0.7 |
| Micronesia, Fed. Sts. | 0.1 | 2,278 | 62.5 | 64.1 | 1.6 | -0.57 | -0.70 | 0.00 | 0.13 | 0.0 |
| Moldova | 4.1 | 569 | 69.1 | 74.2 | 5.1 | 0.15 | 0.01 | 0.00 | 0.14 | 0.0 |
| Mongolia | 2.5 | 663 | 61.4 | 68.0 | 6.6 | 0.27 | 0.00 | 0.00 | 0.27 | 0.1 |
| Montenegro | 0.6 | 2,081 | 73.8 | 75.8 | 2.0 | 0.02 | 0.00 | 0.00 | 0.01 | 0.0 |
| Morocco | 30.5 | 1,454 | 70.0 | 73.2 | 3.2 | 0.28 | 0.02 | 0.00 | 0.26 | -0.1 |
| Mozambique | 18.5 | 291 | 51.9 | 58.3 | 6.4 | 3.24 | 1.36 | 1.37 | 0.42 | 1.3 |
| Myanmar | 47.3 | 149 | 59.6 | 69.3 | 9.7 | 1.55 | 0.32 | 0.26 | 0.96 | 2.8 |
| Namibia | 1.9 | 1,811 | 52.4 | 65.1 | 12.7 | 8.57 | 7.46 | 0.11 | 0.71 | 0.2 |
| Nauru | 0.0 | n.a. | 58.4 | 63.3 | 4.8 | 0.06 | -0.11 | 0.00 | 0.17 | 0.0 |
| Nepal | 24.8 | 276 | 66.0 | 71.0 | 5.0 | 0.46 | -0.03 | 0.01 | 0.48 | 0.3 |
| Netherlands | 16.1 | 29,402 | 78.5 | 81.6 | 3.1 | 0.02 | 0.01 | 0.00 | 0.01 | -0.5 |
| New Zealand | 3.9 | 15,703 | 78.7 | 81.6 | 2.9 | 0.01 | 0.00 | 0.00 | 0.00 | -0.1 |
| Nicaragua | 5.1 | 1,012 | 73.5 | 75.3 | 1.8 | -0.07 | -0.17 | 0.00 | 0.10 | -0.1 |
| Niger | 12.2 | 227 | 52.8 | 62.3 | 9.5 | 1.52 | 0.54 | 0.51 | 0.45 | 1.2 |
| Nigeria | 130.8 | 731 | 54.3 | 64.1 | 9.8 | 2.84 | 0.91 | 1.24 | 0.62 | 12.6 |
| North Macedonia | 2.1 | 1,974 | 71.5 | 74.3 | 2.8 | 0.05 | 0.00 | 0.00 | 0.05 | 0.0 |
| Norway | 4.5 | 42,998 | 79.2 | 82.7 | 3.5 | 0.01 | 0.00 | 0.00 | 0.01 | -0.2 |
| Oman | 2.4 | 8,109 | 69.5 | 73.9 | 4.4 | -0.01 | -0.02 | 0.00 | 0.02 | 0.0 |
| Pakistan | 149.5 | 547 | 61.1 | 65.7 | 4.6 | 0.65 | -0.03 | 0.02 | 0.66 | 5.2 |
| Palau | 0.0 | 8,429 | 66.5 | 67.4 | 0.9 | -0.11 | -0.13 | 0.00 | 0.02 | 0.0 |
| Panama | 3.0 | 4,173 | 77.9 | 79.7 | 1.9 | 0.22 | 0.14 | 0.00 | 0.07 | -0.1 |
| Papua New Guinea | 5.9 | 815 | 62.4 | 64.5 | 2.0 | 0.34 | -0.08 | 0.23 | 0.19 | 0.1 |
| Paraguay | 5.3 | 1,312 | 75.9 | 76.4 | 0.5 | -0.08 | -0.12 | 0.00 | 0.04 | 0.0 |
| Peru | 26.2 | 2,018 | 75.5 | 79.9 | 4.4 | 0.29 | 0.04 | 0.01 | 0.24 | -1.0 |
| Philippines | 83.3 | 1,050 | 70.0 | 71.7 | 1.7 | 0.33 | -0.02 | 0.01 | 0.34 | 0.2 |
| Poland | 38.2 | 5,206 | 74.5 | 78.0 | 3.4 | 0.03 | 0.00 | 0.00 | 0.02 | -1.0 |
| Portugal | 10.6 | 12,928 | 77.3 | 81.5 | 4.2 | 0.20 | 0.16 | 0.00 | 0.03 | -0.4 |
| Puerto Rico | 3.9 | 18,973 | 77.2 | 80.2 | 3.0 | 0.30 | 0.29 | 0.00 | 0.01 | -0.1 |
| Qatar | 0.6 | 30,701 | 71.4 | 76.2 | 4.8 | 0.02 | 0.00 | 0.00 | 0.02 | 0.0 |
| Romania | 22.1 | 2,114 | 71.2 | 75.4 | 4.3 | 0.16 | 0.04 | 0.00 | 0.12 | -0.5 |
| Russian Federation | 148.1 | 2,553 | 65.3 | 72.9 | 7.6 | 0.07 | -0.12 | 0.00 | 0.19 | 1.9 |
| Rwanda | 8.5 | 207 | 52.9 | 68.5 | 15.6 | 6.27 | 3.36 | 1.16 | 1.37 | 1.1 |
| Samoa | 0.2 | 1,597 | 68.9 | 70.5 | 1.6 | -0.03 | -0.09 | 0.00 | 0.06 | 0.0 |
| San Marino | 0.0 | n.a. | 81.5 | 82.0 | 0.4 | 0.01 | 0.01 | 0.00 | 0.00 | 0.0 |
| São Tomé and Príncipe | 0.1 | 553 | 65.7 | 70.8 | 5.2 | 1.60 | 0.00 | 0.00 | 0.12 | 0.0 |
| Saudi Arabia | 21.8 | 8,823 | 70.6 | 74.4 | 3.8 | 0.12 | 0.55 | 0.58 | 0.45 | -0.3 |
| Senegal | 10.4 | 681 | 61.1 | 68.3 | 7.2 | 1.60 | 0.00 | 0.00 | 0.03 | 0.4 |
| Serbia | 9.4 | 2,158 | 72.1 | 75.6 | 3.5 | 0.03 | 0.06 | 0.00 | 0.05 | -0.2 |
| Seychelles | 0.1 | 8,432 | 72.0 | 73.4 | 1.5 | 0.12 | 0.02 | 1.30 | 0.27 | 0.0 |
| Sierra Leone | 4.9 | 252 | 52.5 | 61.6 | 9.1 | 3.10 | 0.24 | 2.36 | 0.44 | 0.5 |
| Singapore | 4.2 | 22,160 | 79.9 | 84.5 | 4.6 | 0.07 | 0.03 | 0.00 | 0.04 | -0.2 |
| Slovak Republic | 5.4 | 4,627 | 74.0 | 77.4 | 3.4 | 0.01 | 0.00 | 0.00 | 0.01 | -0.1 |
| Slovenia | 2.0 | 11,805 | 76.7 | 81.2 | 4.5 | 0.01 | 0.00 | 0.00 | 0.01 | -0.1 |
| Solomon Islands | 0.5 | 797 | 56.6 | 59.1 | 2.4 | 0.37 | -0.05 | 0.36 | 0.05 | 0.0 |
| Somalia | 11.4 | n.a. | 52.0 | 58.3 | 6.3 | 1.30 | 0.32 | 0.32 | 0.64 | 0.8 |
| South Africa | 46.9 | 2,497 | 53.6 | 65.1 | 11.5 | 5.73 | 5.03 | -0.01 | 0.60 | 5.1 |
| South Sudan | 7.6 | n.a. | 57.7 | 63.4 | 5.7 | 1.07 | 0.24 | 0.48 | 0.33 | 0.4 |
| Spain | 42.0 | 17,098 | 79.8 | 82.9 | 3.1 | 0.09 | 0.08 | 0.00 | 0.01 | -1.4 |
| Sri Lanka | 19.1 | 1,027 | 73.3 | 77.2 | 3.9 | 0.13 | 0.01 | 0.01 | 0.11 | -0.5 |
| St. Kitts and Nevis | 0.0 | 10,304 | 70.6 | 72.0 | 1.4 | -0.36 | -0.38 | 0.00 | 0.02 | 0.0 |
| St. Lucia | 0.2 | 5,656 | 73.5 | 75.0 | 1.5 | 0.13 | 0.10 | 0.00 | 0.03 | 0.0 |
| St. Vincent and the Grenadines | 0.1 | 4,328 | 71.6 | 72.8 | 1.3 | 0.42 | 0.40 | 0.00 | 0.02 | 0.0 |
| Sudan | 28.3 | 555 | 63.1 | 70.3 | 7.2 | 0.24 | -0.05 | 0.18 | 0.10 | 0.8 |
| Suriname | 0.5 | 2,872 | 69.3 | 72.5 | 3.2 | 0.93 | 0.50 | 0.41 | 0.02 | 0.0 |
| Sweden | 9.0 | 29,846 | 80.2 | 82.6 | 2.5 | 0.01 | 0.00 | 0.00 | 0.01 | -0.2 |
| Switzerland | 7.4 | 42,808 | 80.7 | 83.7 | 3.0 | 0.03 | 0.03 | 0.00 | 0.01 | -0.2 |
| Syrian Arab Republic | 17.4 | 1,305 | 72.7 | 73.7 | 1.1 | 0.01 | 0.00 | 0.00 | 0.01 | -0.1 |
| Taiwan, China | 22.5 | 13,651 | 77.5 | 80.1 | 2.6 | 0.09 | -0.01 | 0.00 | 0.10 | -0.6 |
| Tajikistan | 6.6 | 188 | 66.9 | 69.5 | 2.5 | 0.25 | 0.03 | 0.03 | 0.19 | 0.0 |
| Tanzania | 36.2 | 382 | 54.3 | 67.0 | 12.7 | 9.02 | 7.09 | 1.06 | 0.50 | 3.9 |
| Thailand | 63.6 | 2,094 | 72.9 | 78.1 | 5.2 | 1.06 | 0.82 | 0.01 | 0.23 | -2.4 |
| Timor-Leste | 0.9 | 509 | 65.7 | 70.4 | 4.8 | 1.16 | 0.27 | 0.36 | 0.52 | 0.0 |
| Togo | 5.1 | 443 | 56.0 | 64.9 | 8.8 | 4.29 | 3.26 | 0.54 | 0.40 | 0.4 |
| Tonga | 0.1 | 1,829 | 71.0 | 72.9 | 1.9 | 0.04 | -0.01 | 0.00 | 0.05 | 0.0 |
| Trinidad and Tobago | 1.3 | 7,163 | 71.1 | 74.7 | 3.6 | 0.58 | 0.57 | 0.00 | 0.01 | 0.0 |
| Tunisia | 10.1 | 2,346 | 74.5 | 77.6 | 3.1 | 0.00 | -0.02 | 0.00 | 0.02 | -0.2 |
| Turkey | 70.7 | 3,617 | 75.0 | 78.2 | 3.2 | 0.06 | 0.00 | 0.00 | 0.06 | -1.8 |
| Turkmenistan | 4.3 | 1,880 | 66.4 | 70.7 | 4.3 | 0.26 | 0.03 | 0.00 | 0.23 | 0.0 |
| Tuvalu | 0.0 | 1,662 | 65.0 | 67.9 | 2.8 | -0.03 | -0.14 | 0.00 | 0.11 | 0.0 |
| Uganda | 25.9 | 362 | 50.7 | 66.0 | 15.4 | 10.51 | 6.83 | 2.09 | 0.82 | 3.8 |
| Ukraine | 48.9 | 885 | 68.0 | 70.1 | 2.0 | -0.01 | -0.07 | 0.00 | 0.07 | 0.2 |
| United Arab Emirates | 3.6 | 32,791 | 69.3 | 73.8 | 4.5 | -0.01 | -0.03 | 0.00 | 0.02 | 0.0 |
| United Kingdom | 60.1 | 30,032 | 78.3 | 80.9 | 2.6 | 0.01 | 0.00 | 0.00 | 0.00 | -1.6 |
| United States | 286.2 | 37,971 | 77.0 | 78.7 | 1.7 | 0.08 | 0.08 | 0.00 | 0.00 | -4.2 |
| Uruguay | 3.3 | 4,425 | 74.9 | 77.4 | 2.5 | 0.02 | 0.00 | 0.00 | 0.01 | -0.1 |
| Uzbekistan | 25.9 | 455 | 64.9 | 68.3 | 3.4 | 0.26 | 0.00 | 0.00 | 0.25 | 0.3 |
| Vanuatu | 0.2 | 1,313 | 63.5 | 65.4 | 1.9 | 1.12 | -0.07 | 1.09 | 0.09 | 0.0 |
| Venezuela, RB | 24.1 | 3,778 | 73.7 | 74.8 | 1.1 | -0.05 | -0.01 | -0.07 | 0.03 | -0.2 |
| Vietnam | 82.2 | 547 | 72.4 | 74.3 | 2.0 | 0.41 | 0.01 | 0.03 | 0.37 | -0.9 |
| Zambia | 10.5 | 376 | 45.7 | 63.1 | 17.4 | 11.09 | 8.78 | 0.69 | 0.87 | 2.0 |
| Zimbabwe | 12.1 | 923 | 46.4 | 60.9 | 14.5 | 14.8 | 13.93 | 0.42 | 0.10 | 2.0 |
